# Complete Genome Sequence of *Desulfovibrio desulfuricans* Strain G11, a Model Sulfate-Reducing, Hydrogenotrophic, and Syntrophic Partner Organism

**DOI:** 10.1128/genomeA.01207-17

**Published:** 2017-10-26

**Authors:** Cody S. Sheik, Jessica R. Sieber, Jonathan P. Badalamenti, Kendall Carden, Adam Olson

**Affiliations:** a Department of Biology, University of Minnesota–Duluth, Duluth, Minnesota, USA; b Large Lakes Observatory, University of Minnesota–Duluth, Duluth, Minnesota, USA; c Department of Microbiology and BioTechnology Institute, University of Minnesota, Saint Paul, Minnesota, USA

## Abstract

Here, we report the draft genome of the Gram-negative, sulfate-reducing bacterium *Desulfovibrio desulfuricans* strain G11. Isolated from a rumen fluid enrichment, this culture has been a model syntrophic partner due to its metabolic flexibility. The assembly yielded a single circular chromosome of 3,414,943 bp and a 57% G+C content.

## GENOME ANNOUNCEMENT

*Desulfovibrio* sp. strain G11 was isolated from a rumen fluid enrichment and showed the ability to utilize lactate, ethanol, formate, and H_2_ as growth substrates in the presence of sulfate (1, 2). As an oxidizer of H_2_, strain G11 was used in coculture to aid the isolation and characterization of *Syntrophomonas wolfei* (3). Since this initial usage as a syntrophic partner, G11 has been used by multiple laboratories as a model organism (3–5) due to its ability to utilize H_2_ and formate for interspecies electron transfer (6). Despite its long history in syntrophy research, this microorganism has not been fully characterized. Using the newly sequenced genome, the phylogeny of the 16S rRNA genes and the *dsr*AB proteins places G11 within the *Desulfovibrio* genus and as a member of the species *D. desulfuricans*. Thus, we suggest the name be *D. desulfuricans* strain G11.

An axenic culture of G11, obtained from the DSMZ culture collection (DSM number 7057) was grown overnight in anaerobically prepared medium (2) containing 20 mM lactate, 20 mM sulfate, and N_2_:CO_2_ (80:20) headspace. High-quality DNA was extracted using chemical lysis, followed by cleaning with chloroform-isoamyl alcohol and overnight DNA precipitation with linearized polyacrylamide, NaCl, and 100% ethanol. DNA was prepared for sequencing using a 15-kb target insert and sequenced using the PacBio RS II platform at the Mayo Clinic Bioinformatics Core (Rochester, MN, USA). Sequencing was done with two single-molecule real-time (SMRT) cells that generated 169,062 raw reads totaling approximately 700 Mb. *De novo* assembly was performed with the hierarchical genome-assembly process (HGAP) version 3 pipeline in SMRTPortal (7) with the longest subreads to provide 100× coverage. The assembly generated a single circular chromosome verified by dot plotting (Gepard version 1.30). Self-complementary ends were trimmed manually, and the contig was reoriented to begin immediately upstream of *dnaA*, as predicted by Prokka version 1.11 (8). Successive rounds of polishing with Quiver (7) produced a single circular 3,414,943-bp (~170× coverage) chromosome with a G+C content of 57%. NCBI’s Prokaryotic Genome Annotation Pipeline predicted a total of 3,099 genes, 2,892 protein-coding genes, and 3 rRNA operons.

As a model hydrogenotrophic partner, G11 can grow solely on H_2_ and SO_4_. A total of seven hydrogenases were identified in the genome. Of the seven hydrogenases, five are predicted to be NiFe hydrogenases, one is an Fe-only ferredoxin-dependent hydrogenase, and one is an Ech-type hydrogenase. Of the NiFe hydrogenases, three appear to be membrane bound. There are three formate dehydrogenases, none of which contain signal peptides, and only one has a single predicted transmembrane-spanning helix. G11 has an 11-subunit NADH dehydrogenase, complex 1, and an electron transport chain most similar to that of *D. desulfuricans* ATCC 27774 (9, 10). A full repertoire of sulfate-reducing genes were present. Genomic evidence for a full Wood-Ljungdahl pathway, lactate dehydrogenase, and ethanol dehydrogenase confirms axenic growth.

### Accession number(s).

The assembled chromosome has been deposited in GenBank under accession number CP023415 and in the Joint Genome Institute Integrated Microbial Genomics database (2700988708). PacBio reads have been deposited to the Sequence Read Archive (SRX3185062).
